# Does prolonged postpartum insusceptibility undercut the impact of postpartum family planning programming in Sub-Saharan Africa?

**DOI:** 10.1186/s12978-025-02056-4

**Published:** 2025-07-22

**Authors:** Sarah Eustis-Guthrie, Benjamin Williamson, Iqbal Shah

**Affiliations:** 1 Maternal Health Initiative, London, United Kingdom; 2 Harvard T.H. Chan School of Public Health, Boston, USA

**Keywords:** Postpartum family planning, Unmet need, Sub-Saharan Africa, Postpartum insusceptibility

## Abstract

Integrating family planning into postpartum care at health facilities is a well-regarded health intervention recommended by major organizations in the sexual and reproductive health space. However, several recent studies have found limited to no effects on rates of short-spaced pregnancies associated with these interventions. This paper will investigate the role of prolonged postpartum insusceptibility to pregnancy due to abstinence and breastfeeding alongside other factors in reducing the impact of increased postpartum contraceptive use, ultimately concluding that these factors may substantially reduce the efficacy of postpartum family planning programming at reducing unintended short-spaced pregnancies.

## Introduction

Too many women continue to lack autonomy over their reproductive lives: recent estimates suggest that 218 million women worldwide have an unmet need for family planning - defined as the percentage of women of reproductive age who want to space or limit their pregnancies but are not using contraception [1]. Furthermore, complications of pregnancy and childbirth are a leading cause of preventable deaths among both mothers and children, accounting for 287,000 maternal deaths alone in 2020 [2]. The risk is particularly high in sub-Saharan Africa, which accounts for more than two-thirds of global maternal deaths [2]. Short-spaced pregnancies contribute to the burden of maternal and child mortality: birth intervals shorter than 18 months increase neonatal mortality by 57%, while intervals of 6 months or shorter increase maternal mortality by 28% [3, 4]. The prevalence of short birth intervals (defined as inter-birth interval length of fewer than 24 months), and the health impacts associated with these, varies significantly between countries in sub-Saharan Africa. Given that levels of contraceptive use are particularly low in the postpartum period, the promotion of family planning uptake has been identified as a key intervention to reduce the incidence of unintended and short-spaced pregnancies [5, 6]. In response, extensive postpartum family planning programming - interventions that focus specifically on increasing contraceptive uptake in the post-birth period - has been rolled out at health facilities around the world, including more than 60 projects across 28 countries [7]. 

However, recent studies have called the efficacy of facility-based postpartum family planning programming into question in contexts where postpartum insusceptibility is prolonged by abstinence and breastfeeding. Where past studies only measured program effects on contraceptive uptake, taking for granted that increased uptake would translate into reduced unintended and short-spaced pregnancies, more recent studies have put that assumption to the test. In a review of available literature, we identified only three studies measuring the effect of postpartum family planning uptake on rates of unintended and short-spaced pregnancies. The results of these studies raise serious concerns: two found no effect [8, 9], and one found only a 0.7% decrease in short-spaced pregnancies even though rates of contraceptive usage increased [10]. While further studies are needed to substantiate these conclusions, the available literature suggests that facility-based programs may have limited to no effect on reducing unintended short-spaced pregnancies despite increasing contraceptive uptake.

What could cause these unexpected results? One possibility is postpartum insusceptibility to pregnancy, which is common globally and particularly prolonged in sub-Saharan Africa, where a substantial portion of postpartum family planning programming occurs. A significant proportion of sub-Saharan African women in their first year postpartum are unlikely to become pregnant due to abstinence or insusceptibility from breastfeeding. However, the standard approach to unmet need classifies many of these women as nonetheless possessing a need for contraception [11]. While some authors have questioned how well the “unmet need” framework applies in the postpartum period, particularly in regions with high levels of prolonged insusceptibility to pregnancy, this work has received limited engagement [12]. This article will investigate the scope and implications of postpartum insusceptibility in sub-Saharan Africa in order to reevaluate the efficacy of postpartum family planning as a health intervention.

### Postpartum insusceptibility reduces the marginal protection against pregnancy provided by contraceptive use

Increases in family planning uptake are primarily valuable insofar as they empower women to prevent mistimed or unintended pregnancies. The wide range of benefits associated with contraceptive use — including improved empowerment [13], reduced maternal and child mortality [14] labor force participation and increased earnings [15] — are all predominantly derived from reductions in unintended and short-spaced pregnancies. This includes most of the benefits to autonomy, which result from the greater control they afford women over their reproductive lives. Programs that have little effect on rates of unintended pregnancy therefore provide only limited benefits to recipients.

While many programs have increased contraceptive use, only a handful have measured changes to pregnancy rates. Of three facility-based postpartum family planning programs that have measured changes to pregnancy rates, two found no change, while the third found only a small reduction in the incidence of pregnancy [8–10]. Interestingly, both studies that found no change to pregnancy rates – an IUD-focused program in Tanzania and a broader program in Burkina Faso – nonetheless had higher rates of long-acting reversible contraceptive (LARC) uptake among the intervention group [8, 9]. Guo et al. found that an IUD-focused program in Nepal reduced the risk of pregnancy by only 0.7% points [10]. Taken together, these studies suggest that facility-based postpartum programming – the primary type of postpartum family planning programming – may not consistently reduce short-spaced pregnancies.

Given the small number of studies we identified that measure effects on short-spaced pregnancies, we cannot draw sweeping conclusions. However, the results of these studies suggest that further research is needed in order to substantiate the effects of postpartum family planning programming on short-spaced pregnancies. The causes for the limited effects on pregnancy rates found in these studies are unclear, but several hypotheses are worth considering. First, it is highly likely that prolonged abstinence and amenorrhea among many postpartum women significantly reduce the additional protection against short-spaced pregnancy conferred by modern methods for some women. Long durations of postpartum insusceptibility – that is, when the risk of pregnancy is zero or extremely low following a birth – are common in many low- and middle-income countries, particularly those in sub-Saharan Africa. The mean duration of postpartum insusceptibility across the 39 sub-Saharan African countries with recent Demographic and Health Survey (DHS) data is 14.2 months (Table 1) [16]. Postpartum insusceptibility results from the combination of two factors: sexual abstinence and amenorrhea due to breastfeeding.

**Table 1:** Average duration of postpartum insusceptibility, abstinence, and amenorrhea across sub-Saharan African countries

Region	Country	Year of Survey	Duration of Postpartum Insusceptibility (Mean)	Duration of Postpartum Insusceptibility (Median)	Duration of Postpartum Abstinence (Mean)	Duration of Postpartum Amenorrhea (Mean)
Africa			**14.2**	**11.6**	**8.2**	**11.3**
Eastern Africa			**14.8**	**12.2**	**7.1**	**11.8**
	Burundi	2016-17	18.5	17.8	4.8	11.3
	Comoros	2012	9	4.3	4.8	10.4
	Eritrea	2002	17	14.6	7.1	9.4
	Ethiopia	2016	17.5	16	6.3	7.3
	Kenya	2022	11.7	7	7.4	14.6
	Madagascar	2021	12.8	9	7.7	11.8
	Malawi	2015-16	15.8	12.8	8.9	12.5
	Mozambique	2011	17.4	15	13.4	17.5
	Rwanda	2019-20	17.6	15.6	5.4	8.8
	Tanzania	2022	13.1	10.2	7	14.1
	Uganda	2016	13.3	10.9	6.4	13.5
	Zambia	2018	14.3	10.8	8	10.8
	Zimbabwe	2015	14.2	14.2	5.7	11.7
Middle Africa			**14.0**	**11.7**	**8.2**	**10.6**
	Angola	2015-16	15.9	13.8	9.3	7.8
	Cameroon	2018	13.4	10.9	8.2	9.4
	Central African Republic	1994-95	17.5	16.4	12.7	11
	Chad	2014-15	16.1	14.6	5.7	12.5
	Congo	2011-12	11.9	9.9	7.5	5.7
	Congo Democratic Republic	2013-14	14.7	12.4	7.8	13.1
	Gabon	2019-21	11.2	8.2	9.5	11.1
	Sao Tome and Principe	2008-09	11.6	7.2	5	13.8
Southern Africa			**14.73**	**10.33**	**10.03**	**10.75**
	Eswatini	2006-07	15.4	10.7	8.1	11
	Lesotho	2014	14.3	11.2	10.7	7.1
	Namibia	2013	15	11.3	11.4	15
	South Africa	2016	14.2	8.1	9.9	9.9
Western Africa			**15.2**	**13.2**	**10.0**	**11.5**
	Benin	2017-18	14.1	12.2	9.1	10
	Burkina Faso	2021	15.4	13.1	9.4	16.1
	Cote d’Ivoire	2021	13.5	11.3	9.6	12.8
	Gambia	2019-20	14.8	12	9.5	6.9
	Ghana	2022	13.4	9.6	9.2	11.2
	Guinea	2018	21.1	21	19.7	13.8
	Liberia	2019-20	16.3	14.1	14.3	13.2
	Mali	2018	13.2	10.5	6.1	10.3
	Mauritania	2019-21	13.9	10.4	8.4	8.9
	Niger	2012	14.7	14.3	4	7.6
	Nigeria	2018	14.8	12.7	6.2	15.7
	Senegal	2019	15	13.7	7.7	10.4
	Sierra Leone	2019	18.7	17.3	17.7	11.7
	Togo	2013-14	14.5	12.5	9.5	12.1

Data collected from DHS surveys in 39 sub-Saharan African countries from 1988 to 2022

The bolded values indicate averages across a region

In sub-Saharan Africa, postpartum abstinence is commonly practiced for a substantial period of time post-birth, with an average duration of 8.7 months across 39 countries (Table 1) [16]. There is substantial variation within and across countries, with average durations of postpartum abstinence ranging from 4 months (Niger) to 19.7 months (Guinea). Many areas exhibit strong cultural norms against sexual activity in the postpartum period, with abstinence promoted as protecting the health of children and mothers [17–19]. In some contexts, abstinence is encouraged through practices such as the new mother staying with her parents or in-laws for a temporary period post-birth [20]. Notably, the average duration of postpartum abstinence has been on the decline over the past several decades in many countries, but levels remain high [20]. 

While long durations of abstinence contribute to postpartum insusceptibility, amenorrhea due to breastfeeding, also known as lactational amenorrhea, is a more significant factor. Amenorrhea – the absence of a menstrual period – occurs among breastfeeding postpartum mothers for a significant period post-birth. Amenorrhea delays the return of ovulation, a biological necessity for pregnancy [21]. It is believed that the suckling action inhibits the release of the hormone estradiol, which triggers the return of ovulation [22]. As a result of widespread breastfeeding, postpartum amenorrhea is common in sub-Saharan Africa, with an average duration of 11.3 months (Table 1) [16]. 

Lactational amenorrhea as a contraceptive method – commonly known as LAM – was formalized by the Bellagio Consensus, a 1988 gathering of experts in family planning. Drawing on the existing literature, it established the three criteria for LAM: amenorrhea, exclusive breastfeeding with frequent feeds, and the 6-month post-birth cutoff. Yet the formal statement issued by the conference also suggested that this strict definition may not always be appropriate, stating that extending the duration of LAM may be appropriate in regions with long durations of breastfeeding [23]. 

Since 1988, the strict definition established by the Bellagio Consensus has been operationalized, with very little programmatic attention given to its suggestion that LAM may continue to be effective past 6 months in regions with extended periods of breastfeeding. However, several studies have substantiated the claim that pregnancy rates amongst “extended” LAM users at 12 months postpartum are low (Table 2) [24–29]. Indeed, four of the five studies report 12-month efficacy rates over 91%, with only one study reporting a lower rate of 82.8% [29].

**Table 2:** Effectiveness of LAM and extended LAM at preventing pregnancy

Study	6-month efficacy	12-month efficacy
Labbok et al. 1997 [24]	98.0%	91.2%
Ramos et al. 1996 [25]	98.5%	97.4%
Kennedy et al. 1992 [26]	99.3%	94.1%^*^
Kazi et al. 1995 [27]	99.4%	98.9%^*^
Short et al. 1991 [28]	98.3%	93.0%
Tiwari et al. 2018 [29]	100.0%	96.0%

Data collected at 6- and 12- months postpartum from six studies

^*^indicates only amenorrheic women

Several studies suggest that breastfeeding may not need to be exclusive to confer a high level of protection against pregnancy. Studies of LAM use have typically found similarly high rates of protection against pregnancy amongst both “correct” and “incorrect” LAM users, suggesting that postpartum breastfeeding provides a high level of protection by prolonging amenorrhea even when it is non-exclusive. For example, a 1997 study across 10 countries found effectiveness rates of 98.5% and 98.3% among correct and incorrect LAM users, respectively, at 6 months [24]. It is unsurprising that rates of pregnancy would be low amongst amenorrheic women, and indeed the DHS classifies women as insusceptible if she is either amenorrhoeic or abstaining from intercourse (or both), with no consideration of the time that has elapsed since the birth.

Broadening LAM criteria to encompass amenorrheic women at 12 months postpartum who are breastfeeding in any form would more accurately reflect the effectiveness rates of such methods while reducing the burden on healthcare providers. Fully exclusive breastfeeding is demanding for mothers, and only nutritionally recommended for 6 months postpartum [30]. Given the relatively high levels of protection against pregnancy provided by non-exclusive breastfeeding – with effectiveness rates comparable to those of contraceptive pills as actually used – investing substantial resources into improving strict LAM adherence under the current definition appears suboptimal [31]. 

Extended LAM does come with some risk: some, but not most, women ovulate prior to the return of their menses postpartum [32]. As such, if women wait for the return of their menses to obtain contraceptive protection, there is a chance of fertilization in the approximately two weeks between ovulation and menstruation for the minority of women who ovulate prior to menstruation. However, this risk is low and further reduced by the hypothesis that due to breastfeeding’s effects on corpus luteum function, it may take several menstrual cycles before a pregnancy can be supported [22]. 

Given the extent and magnitude of postpartum insusceptibility, why do so many African countries report high levels of unmet need in the postpartum period? The answer is simple: standard definitions of unmet need and contraceptive use minimize the protection provided by postpartum abstinence and amenorrhea and therefore overestimate unmet need for contraception [12]. Among postpartum amenorrheic women, unmet need is calculated based on whether the occurrence and timing of a woman’s most recent pregnancy were intended, rather than her current intentions, and amenorrhea is not considered a form of protection against pregnancy [11]. Hence, some women who currently have a low risk of pregnancy due to amenorrhea are nonetheless classified as possessing unmet need.

Cleland et al. outline an alternative, the “current-status” approach, under which postpartum women who are amenorrheic or abstinent are classified as having no unmet need for contraception [12]. DHS estimates of unmet need among African countries are 2–7 times higher than those using their “current status” definition. For example, DHS statistics from Ghana suggest that 50.6% of postpartum women have unmet need, while the current status approach indicates that 10.9% of postpartum women are at risk. High levels of unmet need amongst postpartum women are a key rationale for prioritizing postpartum family planning; given that these levels seem substantially lower, the case for its importance is correspondingly weaker [6]. 

### Evaluating the value of postpartum family planning programming

Let us return to the question of why several postpartum family planning programs find minimal impact on pregnancy rates despite increasing uptake of modern contraception. As we have discussed, widespread insusceptibility likely plays a key role, reducing the marginal protection against pregnancy provided by modern methods. If an extended LAM user begins using an implant, for example, her risk of pregnancy will likely only be reduced by 5–10% points [24–29, 31]. The three studies that found limited to no effects on pregnancy rates all targeted countries – Burkina Faso, Tanzania, and Nepal – with relatively prolonged postpartum insusceptibility: 15.4, 13.1, and 10.9 months, respectively (Table 1) [16].

One possibility is that the increased uptake produced by many postpartum programs primarily serves to bring forward contraceptive uptake amongst intended future users to a point where they are already protected by prolonged abstinence or amenorrhea, rather than creating entirely new users. This argument is bolstered by evidence from Tran et al. [33], where contraceptive uptake among the intervention group remained similar at 1 and 2 years postpartum, while uptake in the control group substantially increased from year 1 to year 2 [9]. The study’s authors hypothesize that increased uptake among the control group resulted from other contraceptive programming that was occurring; regardless, it brings the marginal value of the facility-based programming into question. Modern contraceptive use is typically low in the early postpartum period and subsequently rises even without additional programming [16]. Given this, the value of programming that merely increases uptake in that early postpartum period when many women are naturally protected by amenorrhea, and thus does not affect rates of pregnancy, may be low.

Postpartum insusceptibility alone cannot fully explain these results, however. While prolonged postpartum insusceptibility is common in many countries, it is certainly not universal; indeed, the continued persistence of short-spaced pregnancies attests to this fact. One possibility is that facility-based postpartum family planning programming may not be increasing rates of contraceptive use among women with the highest risk of short-spaced pregnancies. While one-quarter of births are short-spaced, it appears likely that those at high risk of short-spaced pregnancies are both less likely to be present at facilities and less likely to use contraception: short-spaced births are associated with lower levels of education and income, and poorer and less educated women are typically both less likely to obtain maternal and newborn care at health facilities and less likely to use contraception [34–36]. As a result, facility-based postpartum family planning programs may fail to reach their intended target population, primarily increasing uptake amongst women who are at low risk of a short-spaced pregnancy.

More broadly, historical data suggests that significant increases in contraceptive uptake are not consistently associated with reductions in short-spaced birth frequency, suggesting that increases in contraceptive uptake will not always reduce the incidence of short-spaced pregnancies [34, 37]. Indeed, rates of short-spaced pregnancy in some high-income countries are not significantly lower than in regions such as Sub-Saharan Africa despite much higher rates of contraceptive use. For instance, 18.7% of births from 2015 to 2019 were within 24 months of the previous birth in the United States [38]. Taken together, the evidence suggests that rates of reduction in short-spaced pregnancies cannot simply be extrapolated from increased contraceptive uptake; the situation is substantially more complex. Further studies measuring the effects of facility-based postpartum family planning programming on rates of pregnancy are urgently needed.

While more research is needed, community-based postpartum family planning interventions in Bangladesh and Malawi found substantial reductions in short-spaced pregnancies, reducing the rate of short-spaced pregnancies by 43.5% and 19% respectively [39, 40]. The reason for their greater success in reducing pregnancy rates is unclear, but it is likely that community-based interventions reach those at high risk of short-spaced pregnancies more effectively by including women who do not give birth at health facilities. Furthermore, their significantly heavier-touch approach may play a role; both programs included 5–6 home visits by family planning counselors across the immediate and extended postpartum period. The in-depth engagement with clients afforded by these programs, in contrast with the lighter touch approach often available at health facilities, may lead to a higher level of contraceptive use among those at high risk of short-spaced pregnancies.

While these results are promising, substantial postpartum family planning programming is facility-based rather than community-based and as such there should be significant concerns whether these programs are translating into reductions in short-spaced pregnancies [5, 6]. Additionally, facility-based programs are convenient to implement since family planning can be integrated into postnatal appointments women are also likely to be attending. Community-based programming generally cannot be integrated into existing services as easily or cheaply, likely reducing the ability for this kind of programming to produce a comprehensive reduction in unintended pregnancies. As facility-based programs receive a significant proportion of the attention and funding in the postpartum space, further studies evaluating the effects of such programming on pregnancy rates would be highly beneficial. In doing so, we can enhance efforts to reduce the prevalence of unintended and short-spaced pregnancies, and their associated health impacts.

In areas with long-lasting postpartum insusceptibility, almost all of the women at postnatal care appointments, and a substantial fraction of those at immunization sessions, may be at minimal risk of pregnancy. As a result, modern contraceptive uptake at these touchpoints may have minimal short-term impact for those at postnatal care, and short-term impact for only a minority of women at immunization sessions. For those who continue their contraceptive use, protection from pregnancy will emerge as insusceptibility wanes. This will take an average of 14 months for women accepting immediate postpartum family planning and around 9 months for women attending immunization sessions (assuming the average duration postpartum at immunization sessions is 4.5 months) [16]. 

A further concern is that some users will likely discontinue their use. There is a 36.6% average 1-year contraceptive discontinuation rate across 30 sub-Saharan African countries with DHS data available [16]. While most women will continue use beyond 1 year, this level of discontinuation undercuts the relative value of postpartum family planning compared to other approaches that are also likely to affect pregnancy rates in the first year of use. Furthermore, multiple studies evaluating postpartum family planning interventions found that effects on contraceptive use observed at 1-year post-intervention dissipated by 2 years post-intervention [9, 41]. 

Given concerns about method discontinuation, prioritizing long-acting reversible contraception (LARC) could be more promising since rates of LARC discontinuation are substantially lower [42]. However, “LARC-first” programs raise issues concerning the potential for coercion and a lack of informed choice. Many studies have highlighted bias and coercion associated with some LARC-first counseling in high-income countries [43–46]. Preliminary studies in low- and middle-income countries raise significant concerns, including providers giving misleading and inaccurate information concerning LARCs, or in some cases refusing to remove implants or IUDs, in opposition to women’s preferences [47]. More research in this area is urgently needed. In the postpartum period, IUD insertion immediately post-birth raises ethical concerns, as women may be particularly vulnerable at this time given the stresses of birth. Practices such as offering IUDs during labor without counseling on other methods should therefore be seen as antithetical to free and informed choice [48]. 

There is certainly value in providing family planning guidance in the postpartum period. However, given that short-spaced pregnancies increase the risk of adverse outcomes for both mothers and children, it is vital to identify interventions that demonstrably reduce their incidence [4, 49]. Healthcare providers in low-resource settings have limited time and resources; “kitchen sink” approaches that include all of the information that could provide any benefit to mother’s risk degrading the overall quality of care. Given that there are other effective approaches to increase contraceptive uptake with fewer weaknesses, shifting resources away from facility-based postpartum family planning in areas of prolonged postpartum insusceptibility may be prudent. Programs such as radio-based mass media campaigns and community engagement through Participatory Learning and Action (PLA) groups show promise for increasing contraceptive uptake among large numbers of women at a low cost without being tied to facility-based care [50, 51]. 

Finally, arguments for postpartum family planning programming that heavily prioritize “modern” forms of contraception over other types minimize culturally specific concerns regarding contraception among many African women. Side effects like inconsistent or excess bleeding may have substantially greater effects on women in many low- and middle-income contexts [52–55]. Cultural practices around inconsistent or excess bleeding can prevent women from participating in a range of regular activities, including prayer, sexual activities, and community engagement [56, 57]. More research is needed to form a robust understanding of the full scope of women’s lived experience of contraceptive side effects. Nevertheless, there is clear evidence that the negative consequences of modern contraceptive use can extend far beyond the medical changes induced. As such, the choice of some women not to use hormonal contraception in order to avoid these consequences is worthy of recognition and support.

## Conclusion

Given the finite resources available for family planning programming, it is essential that they are directed towards effective, impactful programs. Reductions in unintended pregnancies are an essential driver of the benefits to maternal and child health, autonomy, and income associated with increased contraceptive uptake; hence, the three studies discussed in this paper which suggest limited to no reductions to short-spaced pregnancies from postpartum programs, call into question the choice to prioritize postpartum family planning programming over other interventions aimed at increasing access to contraception. These results may be driven by high rates of postpartum insusceptibility, particularly in sub-Saharan Africa. In these contexts, modern contraceptive uptake in the early postpartum period is likely providing minimal additional pregnancy protection for most women. This suggests that postpartum family planning programming that focuses on increasing uptake in the early postpartum period appears to be relatively less effective at empowering women to control their reproductive lives. In areas where rates and durations of sexual abstinence and breastfeeding are very low, postpartum family planning programming may remain an effective and appropriate intervention. Outside of these locations, other approaches – those with more strongly established effects on unintended and short-spaced pregnancies – may be substantially more impactful at increasing women’s autonomy, and therefore may be more promising areas for further investment. While these conclusions are preliminary, given the small number of studies available, they suggest that further research is needed to better understand these programs.

## Data Availability

No datasets were generated or analysed during the current study.
